# BDNF and Pro-BDNF in Amyotrophic Lateral Sclerosis: A New Perspective for Biomarkers of Neurodegeneration

**DOI:** 10.3390/brainsci12050617

**Published:** 2022-05-09

**Authors:** Giulia Riolo, Claudia Ricci, Nicoletta De Angelis, Carlotta Marzocchi, Gisella Guerrera, Giovanna Borsellino, Fabio Giannini, Stefania Battistini

**Affiliations:** 1 Department of Medical, Surgical and Neurological Sciences, University of Siena, 53100 Siena, Italy; giulia.riolo@gmail.com (G.R.); nicoletta.deangelis91@gmail.com (N.D.A.); carlottamarzocchi@libero.it (C.M.); fabio.giannini@unisi.it (F.G.); stefania.battistini@unisi.it (S.B.); 2 Neuroimmunology Unit, Santa Lucia Foundation IRCCS, 00143 Rome, Italy; g.guerrera@hsantalucia.it (G.G.); g.borsellino@hsantalucia.it (G.B.)

**Keywords:** Amyotrophic Lateral Sclerosis, BDNF, Pro-BDNF, biomarkers, disease progression, CSF, serum

## Abstract

Amyotrophic Lateral Sclerosis (ALS) is characterized by the progressive degeneration of upper or lower motor neurons, leading to muscle wasting and paralysis, resulting in respiratory failure and death. The precise ALS aetiology is poorly understood, mainly due to clinical and genetic heterogeneity. Thus, the identification of reliable biomarkers of disease could be helpful in clinical practice. In this study, we investigated whether the levels of brain-derived neurotrophic factor (BDNF) and its precursor Pro-BDNF in serum and cerebrospinal fluid (CSF) may reflect the pathological changes related to ALS. We found higher BDNF and lower Pro-BDNF levels in ALS sera compared to healthy controls. BDNF/Pro-BDNF ratio turned out to be accurate in distinguishing ALS patients from controls. Then, the correlations of these markers with several ALS clinical variables were evaluated. This analysis revealed three statistically significant associations: (1) Patients carrying the *C9orf72* expansion significantly differed from non-carrier patients and showed serum BDNF levels comparable to control subjects; (2) BDNF levels in CSF were significantly higher in ALS patients with faster disease progression; (3) lower serum levels of Pro-BDNF were associated with a shorter survival. Therefore, we suggest that BDNF and Pro-BDNF, alone or in combination, might be used as ALS prognostic biomarkers.

## 1. Introduction

Amyotrophic Lateral Sclerosis (ALS) is a heterogeneous condition resulting from the progressive degeneration of motor neurons in the brain and spinal cord. The classical disease has a focal onset, characterized by the loss of function of upper or lower motor neurons, resulting in no muscle nourishment and leading to inevitable paralysis [1,2]. Importantly, atrophy quickly progresses from the region of onset to close spinal regions and very often the failure of respiratory muscles is fatal for ALS patients [3]. Riluzole and edaravone, the only FDA approved drugs so far, marginally enhance survival and slow the clinical progression of the disease, increasing the urgency for new effective therapeutic treatments [4].

The worldwide incidence of ALS accounts for 1.6/100,000 people each year [5]. Almost 90% of cases are classified as sporadic (sALS) and only the remaining 10% have a family history of disease (fALS), characterized by a Mendelian dominant inheritance with incomplete penetrance [6].

Despite decades of research, the precise aetiology of ALS is definitely poorly understood. Diagnosis, commonly made with a considerable delay from symptom onset, is mostly based on electrophysiological examinations and clinical judgment, because there is not a determinant diagnostic laboratory test for ALS. Phenotypes of ALS patients differ in signs and symptoms, age of onset, clinical progression and duration of illness. The clinical variability is accompanied by a genetic heterogeneity in this pathology. Four major genes have been identified as causative of motor neuron degeneration: *C9orf72* (Chromosome 9 open reading frame 72), *SOD1* (Cu^2+^/Zn^2+^ superoxide dismutase), *TARDBP* (TAR DNA binding protein) and *FUS* (RNA binding protein Fused in Sarcoma) [6,7]. However, only a subset of patients harbors genetic mutations, regardless of ALS form (familial or sporadic), further complicating the diagnosis and the ambiguity concerning disease pathogenesis [8,9].

In order to improve the diagnosis of ALS and resolve the complexities of phenotypic heterogeneity, an important issue may be the identification of reliable molecular “hallmarks” of the disease for use in clinical practice.

In the last years, an increased interest has been shown in considering the brain-derived neurotrophic factor (BDNF) as a promising biomarker of neurodegeneration, which may not only aid in the diagnosis of ALS, but might also predict clinical course and progression of the disease.

Encoded by the *BDNF* gene, which is localized on chromosome 11 in humans [10], the BDNF is well-known to be involved in many neurological events, from neurogenesis to neurodegeneration [11], regulating neuronal growth and morphology, as well as synaptogenesis [12,13,14,15]. It is therefore not surprising that the BDNF expression is abundant and tightly regulated in the Central Nervous System (CNS), but it has been also found in serum, plasma and lymphocytes [14,16].

Like all other proteins of the same family, which includes NGF (Nerve Growth Factor) and several neurotrophins (NT-3, NT-4/5) [4,17], BDNF is the final product of a multistep process of synthesis, which generates several precursor isoforms [18]. This neurotrophic factor is firstly synthesized as Pre-Pro-BDNF, a precursor protein containing a signal peptide for translocation in the endoplasmic reticulum (ER) [19]. The pre-domain is then cleaved into the Golgi apparatus, resulting in the formation of Pro-BDNF (proneurotrophin isoform of BDNF, ~30 kDa) [20], which further undergoes intra- or extra-cellular proteolysis to generate the mature form (BDNF, ~13 kDa). Intra-cellular cleavage of pro-domain sequence may occur in trans-Golgi as well as in intra-cellular vesicles, mediated by furin and convertases, respectively. Instead, plasmin and various matrix metalloproteinases (MMP2, MMP3, MMP7 and MMP9) are responsible for extra-cellular processing of Pro-BDNF [14,15,18].

In both cases, the resultant BDNF is found in the extra-cellular space, where its interaction to a specific membrane receptor will determine the activation of several signaling pathways in neurons. However, proteolysis may be missed and, also, Pro-BDNF can be found in the extra-cellular space as a functionally effective isoform [18].

Interestingly, well-documented evidence has revealed that Pro-BDNF is a bioactive product with its own biological functions, which seem to be different to those of mature BDNF. To date, several studies support a “yin-yang hypothesis”, proving that the two isoforms elicit opposite effects by binding to two distinct receptors [13,14,15,21].

Pro-BDNF targets the p75^NTR^ (p75 neurotrophin receptor), belonging to the TNF (Tumor Necrosis Factor) receptor family, through the mature domain, and sortilin receptor, through the pro-domain sequence. This results in the formation of the Pro-BDNF/p75^NTR^/sortilin binding complex, which is able to initiate several biochemical cascades: the RhoA (Ras homolog gene family member A)-dependent pathway, involved in neuronal growth [22], the NF-κB (Nuclear Factor-kappaB)-linked pathway, which promotes neuronal development and survival [23], and the JNK-related pathway that triggers apoptosis [24,25].

Instead, the mature BDNF interacts preferentially with the Tyrosine kinase B (TrkB) receptor, which consequently undergoes homodimerization and autophosphorylation, and with lower affinity to the p75^NTR^ receptor [23]. Phosphorylated-TrkB receptors translocate to the cell membrane [26] and activate several signalling proteins: PI3K, MAPK, PLC-γ and GTPases. Each of these proteins is involved in regulating different cellular processes: PI3K exerts anti-apoptotic/pro-survival activity; MAPK and GTPases control neuronal cytoskeleton organization, as well as dendritic growth and branching [27]; PLC-γ enhances synaptic plasticity [23]. Briefly, Pro-BDNF mainly influences neuronal death, whereas mature BDNF promotes neuronal survival [28].

However, taken together, it is likely that pro-isoform and mature peptide share important biological functions, and the final effect on neurons seems to derive from a tightly regulated balance between the BDNF/TrkB and Pro-BDNF/p75^NTR^ activities. The balance is maintained by stabilizing proteins through the binding with the pro-peptide: sortilin and HAP1 (Huntingtin-associated protein 1) facilitate the cleavage of pro-domain and promote maturation [29], while SPIG1 (SPARC-related protein containing immunoglobulin domains 1) protein suppresses the transition of Pro-BDNF to mature BDNF [30].

Thus, the BDNF/Pro-BDNF ratio appears to be fundamental, and impairment of this balance may play a major role in the development of nervous system diseases. Moreover, BDNF-TrkB activity is altered in several neurodegenerative diseases, including ALS, and this may suggest a correlation with neuronal damage [4].

The aim of this study is to assess whether BDNF and Pro-BDNF, alone or in combination, may represent biomarkers for ALS. To evaluate how the levels of these neurotrophins may change in different pathological conditions, BDNF and Pro-BDNF were measured in serum and cerebrospinal fluid (CSF) of ALS patients, healthy controls and patients affected by other neurological diseases. In addition, we evaluated the associations of these biomarkers with the presence of the hexanucleotide expansion in the *C9orf72* gene, to unravel possible peculiarities due to genetic asset. Furthermore, we analyzed the associations of BDNF and Pro-BDNF with ALS clinical variables, to determine though they might be used as prognostic biomarkers.

## 2. Materials and Methods

### 2.1. Patients

For the serum analysis, the studied population included 75 ALS patients, consecutively enrolled, and a total of 88 control subjects, divided into three groups: 49 age- and sex-matched individuals from the same ethnic background with no history of neurological diseases (healthy controls); 19 patients diagnosed with Alzheimer’s Disease (AD), chosen as neurodegenerative disorder other than ALS; 20 patients affected by inflammatory diseases (ID), 12 of whom with Multiple Sclerosis (MS) and 8 with a diagnosis of Guillain-Barrè syndrome (GBS), subtype acute inflammatory demyelinating polyneuropathy (AIDP).

For the CSF analysis, the study comprised a subgroup of 34 ALS patients among those enrolled for the serum testing, 11 healthy control subjects (patients who underwent lumbar puncture for microbiological diagnostic purposes and were negative for all the performed tests), 10 AD patients and 19 subjects affected by inflammatory diseases (including subjects enrolled for the serum testing for both the groups). Characteristics of ALS patients and controls are summarized in Table 1.

Written consent for genetic analysis was obtained from each individual. This study was approved by the Local Ethics Committee (Comitato Etico Regionale per la Sperimentazione Clinica della Regione Toscana, sezione Area Vasta Sud-Est-Prot. N. 12784_2018, 16 April 2018) in accordance with the ethical standards of the Declaration of Helsinki.

ALS diagnosis was made according to El Escorial Revisited criteria [31]. Patients diagnosed to have Definite, Probable and Probable laboratory supported ALS were included in the study. Briefly, sites of onset were recorded as spinal vs. bulbar. Age at onset was defined by the onset of first symptoms. Clinical severity was assessed with the ALS Functional Rating scale (ALSFRS-R); respiratory function was estimated by Forced Vital Capacity (FVC). The rate of disease progression (ΔFS) at recruitment was calculated by dividing the difference between maximum ALSFRS-R score and ALSFRS-R score recorded at the time of lumbar puncture and by symptom duration (months), as described by Kimura and colleagues [32]. According to the ΔFS, three rates of progression could be calculated: slow (ΔFS < 0.5), intermediate (ΔFS ≥ 0.5 < 1) and rapid (ΔFS ≥ 1). The survival endpoint was death or time of initiation of invasive ventilatory support. The mean duration of the disease was calculated as the time occurring between onset and survival endpoints. Patients were under observation from the diagnosis until the survival endpoint; for alive patients, the follow up was still ongoing at the time of manuscript writing. All patients were screened for mutations in the major genes associated with the disease (*C9orf72*, *SOD1*, *TARDBP*, FUS). Twelve patients carried the hexanucleotide expansion in the *C9orf72* gene, while no mutations were identified in the other genes. All patients were treated with riluzole, 50 mg taken orally twice daily, none of them was enrolled in clinical trials. There were no significant differences between the subgroup of 34 ALS patients included in the CSF analysis and the total of the 75 ALS patients in the distribution of clinical variables (age at onset, sex, site of onset, genetics, onset-diagnosis period, disease duration).

Clinical features of ALS patients are reported in Table 2.

### 2.2. Serum and CSF Sampling

Serum samples were obtained by standard procedures, aliquoted and stored at −80 °C. For all the ALS patients, blood collection was performed at the follow up visit after one month treatment with riluzole. The mean interval between disease onset and blood collection was 13.1 months (range 5–36 months).

CSF samples were obtained by lumbar puncture, centrifuged (1600× *g*, 4 °C, 15 min), divided into aliquots to use for the different diagnostic and research investigations, frozen within 40 min of collection and stored at −80 °C until use. All procedures from withdrawal to storage of CSF samples were performed according to the Guidelines for CSF Biobanking for Biomarker Research [33]. Lumbar puncture was performed at the time of ALS diagnosis, with a mean interval between first symptoms and diagnosis of 13.5 months (range 5–48 months).

### 2.3. ELISA Assays

BDNF and Pro-BDNF concentrations in serum samples were quantified by enzyme-linked immunosorbent assays (ELISA) (R&D Systems, Minneapolis, MN, USA), in accordance with manufacturer’s instructions. Concentrations were expressed as pg per mL of sample. Limits of detection were 23.4 pg/mL for BDNF and 100 pg/mL for Pro-BDNF.

### 2.4. Simoa Assay

BDNF levels in CSF were measured by digital ELISA using the Simoa^®^ technology (Quanterix, Lexington, MA, USA). Briefly, while traditional ELISA systems require large volumes with consequent dilution of the reaction product and reduction in sensitivity to the picomolar (i.e., pg/mL) range and above, SIngle-MOlecule Analysis (Simoa) provides enhanced sensitivity over conventional ELISA. Analytes are captured in semi-homogenous solution by antibody coated magnetic beads. Beads are trapped in femtoliter sized microcavities, and each molecule generates a signal that can be measured as an “on” (presence) or “off” (absence) signal. This increases the sensitivity of Simoa assays typically by a factor of 100 to 1000 as compared to conventional ELISA. For BDNF, the lower limit of quantification was 0.0297 pg/mL and the dynamic range in CSF was 0–240 pg/mL. No Pro-BDNF measurement kit was available at the time of the study.

### 2.5. Statistical Analysis

All statistical analyses were carried out by using the software package SPSS v13.0. *p*-values smaller than 0.05 were considered statistically significant.

Normality of the data was assessed by the Shapiro–Wilk test. In the case of normal distributions, statistical differences were verified by Student’s unpaired two-tailed t-test or ANOVA. In the case of non-normal distributions, two-tailed Mann–Whitney U test or Kruskal-Wallis test was used.

Receiver operating characteristic (ROC) curves were plotted to evaluate the power of BDNF, Pro-BDNF or BDNF/Pro-BDNF ratio to differentiate ALS patients from controls.

Spearman’s correlation coefficient was used to assess the association between BDNF and Pro-BDNF in serum and CSF.

To evaluate the association of BDNF and Pro-BDNF levels with ALS clinical variables, patients were stratified by sex (males/females), site of onset (spinal/bulbar), presence of *C9orf72* expansion and Body Mass Index (BMI: ≥ or <25), and statistical analyses were performed by Mann–Whitney U-test. For the analysis of correlation with ΔFS, Kruskal-Wallis test and Mann–Whitney U-test were used. Spearman’s rho (*r*) was calculated to find correlations with age at onset. Chi square test or exact Fisher test was used when patients were stratified into subgroups based on marker levels: increase in both markers, increase in BDNF and decrease in Pro-BDNF, decrease in both.

In all the analyses, increase and decrease in BDNF and Pro-BDNF in ALS patients were defined comparing their levels to the median values of the control group.

Associations of BDNF and Pro-BDNF levels with disease duration were estimated using the Kaplan-Meier method and compared by the log-rank. In this case, patients were divided into a high expression group (levels greater than the median) and a low expression group (levels less than the median).

Power analysis showed that our sample had a statistical power >80.0%, assuming a significance level (α) of 5%.

## 3. Results

### 3.1. BDNF and Pro-BDNF in Serum

**Figure 1:**
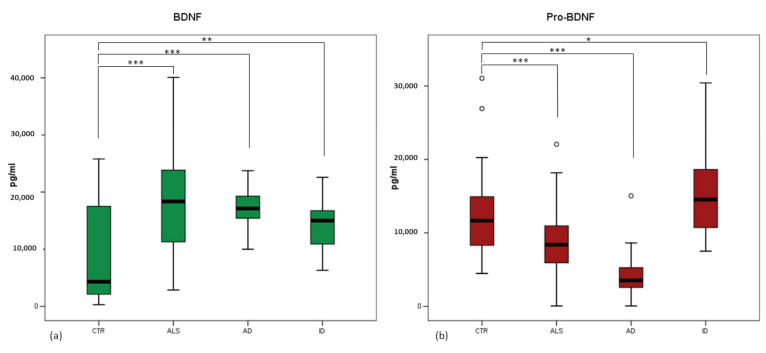
Boxplots showing BDNF (**a**) and Pro-BDNF (**b**) amounts in serum of the three groups of patients (Amyotrophic Lateral Sclerosis, ALS; Alzheimer Disease, AD; Inflammatory Diseases, ID) and healthy controls (CTR). (**a**) BDNF levels are strongly significantly increased in ALS and AD patients and less significantly increased in ID patients, compared to control group. (**b**) Compared to controls, Pro-BDNF is decreased in a significant manner in ALS group and even more in AD group, while Pro-BDNF is increased in ID patients. Black horizontal line: median; o: outliers. *: *p* < 0.05; **: *p* < 0.001; ***: *p* < 0.0001.

BDNF levels were significantly increased in all the three groups of patients compared to healthy controls (Figure 1a). In particular, the difference was highly significant in the case of ALS (controls: 9098.68 ± 1210.76 pg/mL; ALS: 17,235.3 ± 1033.2; *p* < 0.0001), and AD (18,496.00 ± 1165.27; *p* < 0.001) and less in the case of ID (16,180.35 ± 1323.65; *p* = 0.004). There were no statistically significant differences across the three groups of patients (ALS vs. AD: *p* = 0.719; ALS vs. ID: *p* = 0.453; AD vs. ID: *p* = 0.101).

Pro-BDNF serum concentrations significantly differed among the groups of subjects (*p* < 0.0001) (Figure 1b). In this case, there was a statistically significant decrease in ALS patients compared to controls (controls: 12,522.61 ± 885.46; ALS: 9054.79 ± 927.77; *p* < 0.001), even more evident in AD patients (4534.37 ± 757.22; *p* < 0.0001), whereas in ID patients, the concentrations were increased (17,344.85 ± 2830.14; *p* = 0.048). Predictably, the difference between ALS and ID was even more significant (*p* < 0.0001) than in comparison to healthy controls, and the difference between ALS and AD was also significant (*p* < 0.001).

Correlation analysis showed no significant relationship between markers and age in any pathological group as well as in the group of healthy controls. Thus, differences in serum levels of BDNF and Pro-BDNF do not appear to be age-related. Of note, a significant difference between MS and GBS patients was present for Pro-BDNF: MS samples showed Pro-BDNF levels in line with those of control subjects, whereas a significant increase was measured in GBS patients. These results are summarized in Tables S1 and S2, and Figures S1 and S2.

Correlation analysis showed a weak positive correlation between BDNF and Pro-BDNF levels in ALS patients (*p* = 0.022), which was not present in the other groups (CTR: *p* = 0.871; AD: *p* = 0.678; ID: *p* = 0.535).

Given the opposite trend of BDNF and Pro-BDNF in the ALS group compared to healthy controls, the BDNF/Pro-BDNF ratio was considered for further analyses. As shown in Table 3, the differences from the controls were confirmed, and the difference between the ALS and AD was emphasized. These relationships, therefore, could discriminate patients from healthy controls more effectively than the values of individual markers.

Based on these data, the ROC (Receiver Operator Characteristic) curves were elaborated to evaluate the sensitivity and specificity of the markers. The results are shown in Figure S3. As suggested by the previous analysis, BDNF/Pro-BDNF ratio showed the best accuracy, with a value of area under curve (AUC) close to 0.8 (95%; IC: 0.713–0.886). At the cutoff value of 1.40, the optimal sensitivity and specificity were 77% and 67%, respectively.

A more detailed analysis of the data revealed that, while in all sera of AD patients, BDNF was increased and Pro-BDNF decreased compared to controls, in sera of ALS patients, the trend was less homogeneous. Most cases showed the same trend as AD (63.2%), however, in some patients both markers decreased (22.4%) or increased (14.4%). These differences were considered for subsequent statistical analyses.

### 3.2. BDNF in CSF

**Figure 2:**
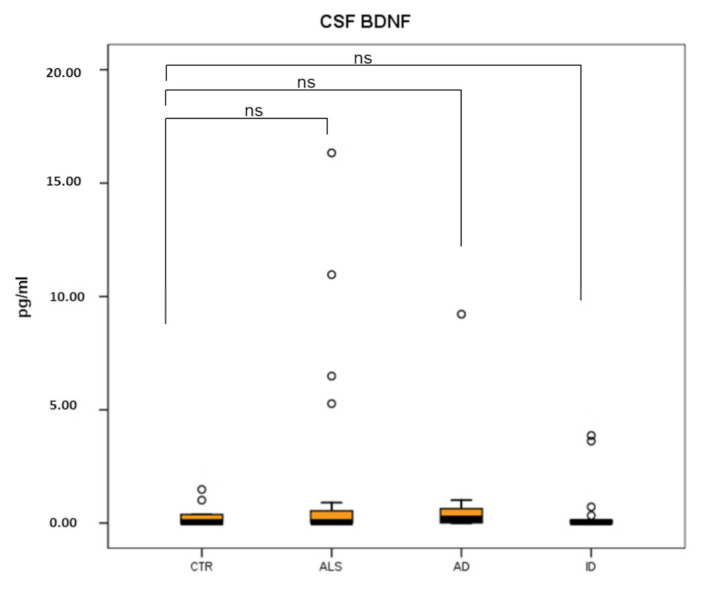
Boxplot showing BDNF levels in CSF of three groups of patients (ALS, AD and ID) and healthy controls (CTR). BDNF levels were not significantly different in ALS patients (*p* = 0.964), AD patients (*p* = 0.604) and ID patients (*p* = 0.711), compared to control subjects. Black horizontal line: median; o: outliers; ns: not significant.

No significant differences in CSF BDNF levels were present between the three groups of patients (ALS: 1.35 ± 0.66 pg/mL; AD: 1.36 ± 1.13 pg/mL; ID: 0.53 ± 0.30 pg/mL) compared to healthy controls (0.32 ± 0.16 pg/mL pg/mL) and among patients’ groups (*p* > 0.05). Results are summarized in Figure 2. Pro-BDNF was undetectable in CSF.

Statistical analysis revealed no correlation between CSF BDNF and serum BDNF or Pro-BDNF levels in any of the patients’ groups.

### 3.3. Correlation with ALS Clinical Variables

Associations of BDNF and Pro-BDNF levels with sex, site of disease onset (spinal versus bulbar), age at disease onset, presence of *C9orf72* hexanucleotide expansion, BMI, ALSFS-R, FVC, progression rate and survival were evaluated.

#### 3.3.1. Serum

BDNF and Pro-BDNF serum levels were analyzed in the ALS patients’ group to evaluate possible correlations with clinical variables. As shown in Table 4, the analysis revealed a significant association between the presence of the hexanucleotide expansion in the *C9orf72* gene and BDNF levels. In this case, mean BDNF levels in expansion-carrying patients were significantly lower than those of non-carrier patients (11,562.09 ± 2825.11 vs. 18,337.12 ± 1079.81). BDNF levels in the *C9orf72* expansion carriers were in line with those of the control subjects (*p* = 0.17068), showing a strong difference from those measured in non-carrier patients (*p* < 0.0001 compared to controls).

In addition, a significant association was observed between Pro-BDNF levels and BMI, with lower Pro-BDNF levels in patients with BMI > 25 than in patients with BMI < 25 (6281.64 ± 1046.85 vs. 11,663.99 ± 2736.50). Pro-BDNF levels in patients with BMI < 25 were of borderline significance (*p* = 0.05486) when compared with the controls, while the significance was evident in the group with BMI > 25 (*p* < 0.001).

Statistical analysis was also performed by stratifying ALS patients into subgroups, based on the trend of the two markers versus controls. Three subgroups were considered: increase in both markers, increase in BDNF and decrease in Pro-BDNF, decrease of both (no patients displayed a decrease in BDNF and increase in Pro-BDNF). The distribution of patients in these subgroups was then analyzed, based on clinical variables. The results are shown in Table 5. In this case, the only significant association was with *C9orf72* expansion. In fact, the patients carrying the expansion showed a distribution of the various subgroups that differed from the non-carriers, with a significant increase in the subjects with a reduction in the levels of both markers (58.4% vs. 14.7%), as shown in Table 5.

**Figure 3:**
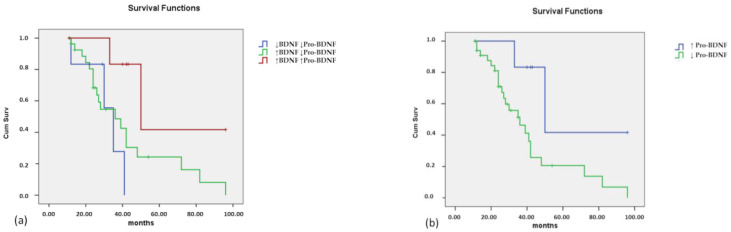
Kaplan-Meier survival curves in relation to BDNF and Pro-BDNF levels in patients not carrying the *C9orf72* expansion. (**a**) Blue line: decrease of both BDNF and Pro-BDNF levels; green line: increased BDNF and decreased Pro-BDNF; burgundy line: increase in both BDNF and Pro-BDNF levels. (**b**) Blue line: increased Pro-BDNF levels; green line: decreased Pro-BDNF (regardless BDNF levels). Censored values (+) indicate the last known follow-up time for those subjects still alive at the time of analysis.

Associations with ALSFS-R an FVC are reported in Tables S3 and S4 of Supplementary file. Considering that patients carrying *C9orf72* expansion displayed BDNF levels not significantly different from those of controls, we decided not to include them in survival analyses. An analysis considering these patients as well is reported in Supplementary File, Figure S5. Patients were divided into the same three groups as in Table 5: decrease in both markers, increase in BDNF and decrease in Pro-BDNF, increase in both markers. The result was not statistically significant (*p* = 0.069), but a tendency to a shorter survival in the presence of decreased levels of Pro-BDNF was shown (Figure 3a). To better understand this trend, patients were divided into two groups, considering all patients with decreased Pro-BDNF levels, regardless of BDNF levels, and comparing them with all other patients. In this case, the result was significant (*p* = 0.029), with a shorter survival in patients with decreased Pro-BDNF levels (Figure 3b).

#### 3.3.2. CSF

We did not find any association between BDNF levels in CSF levels and clinical variables, except for the progression rate (Table 6). In this case, there was a significant association of BDNF levels with fast progression rate of the disease (*p* = 0.026). Of note, all patients with CSF BDNF levels ≥ 0.7 pg/mL belonged to the fast progression group.

**Figure 4:**
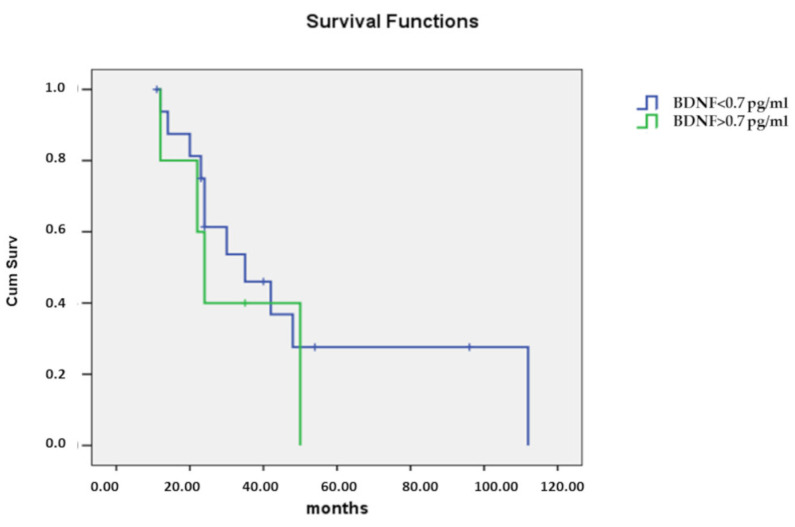
Kaplan-Meier survival curves in relation to CSF BDNF levels. Blue line: BDNF < 0.7 pg/mL; green line: BDNF concentration > 0.7 pg/mL.

In light of these results, survival analysis was performed by stratifying patients into two groups, based on the BDNF levels, using 0.7 pg/mL as a cutoff. The result was not statistically significant, but a trend to a shorter survival in the presence of higher levels of BDNF was observed (Figure 4).

## 4. Discussion

BDNF plays a critical role in maintaining the functionality of the nervous system in both physiological and pathological conditions. The processing of BDNF from Pro-BDNF is important for neuronal development, neuronal survival and synaptic plasticity. Pro-BDNF maturation to BDNF, which happens via intracellular or extracellular proteases, is a delicate balance, which affects the modulation of cell survival or death [34] and seems to be important in controlling BDNF activity in several pathological conditions, including neurodegenerative diseases [35]. Since BDNF can bidirectionally cross the blood-brain barrier, through a high-capacity, saturable transport system [36,37], its levels in both brain and serum are supposed to reflect neurological insults or pathological conditions in patients.

Despite increasing evidence for a critical role of BDNF in motor neuron survival [38,39,40,41], to our knowledge, only two studies have measured BDNF in serum [42,43] and CSF [42,44] of ALS patients so far, failing to find significant differences with control groups. In our study, BDNF levels in CSF did not display any significant differences among ALS, AD, ID patients and healthy controls, confirming previous observations. On the other hand, our research showed serum BDNF concentrations significantly higher in ALS patients than in healthy controls. A similar BDNF increase was also measured in AD and ID, without differences with ALS. The discrepancy with the results of the other studies may be explained considering that our work has been carried out in a larger cohort of ALS patients in comparison to those previously performed. Similar conflicting results have been also reported in the studies on circulating BDNF levels in AD patients. Some studies described a decrease in peripheral BDNF levels [45,46,47], whereas others found no difference or an increase in BDNF concentrations in AD patients [48,49,50]. It has been hypothesized that the reduction in serum BDNF levels is typical of late-stage disease, while in the early stage of AD, BDNF levels increased as a neuroprotective strategy in response to various insults [51,52]. The same compensatory increment of BDNF synthesis in the first stages of the disease could be responsible for the increased BDNF levels measured in ALS patients.

Moreover, an important issue to consider is the kind of assay used in the various studies to quantify these neurotrophins. The antibody-specificity in recognizing only the mature form of BDNF or only the Pro-BDNF form is essential to obtain comparable results. However, the availability of antibodies able to distinguish between the two forms is relatively recent. This generated an experimental heterogeneity among previous studies, which makes the comparisons of results quite difficult.

In this study, for the first time, to the best of our knowledge, we have also measured Pro-BDNF levels in serum of ALS patients and compared them to the control groups. In this case, a significant decrease in pro-BDNF was measured in ALS patients in comparison to healthy controls, a feature shared also by AD patients. On the contrary, Pro-BDNF levels were significantly increased in patients belonging to the ID group. Of note, in this case, the Pro-BDNF increase was essentially due to the GBS patients, while the concentrations in MS patients were in line with those of healthy controls. These data suggest that Pro-BDNF, more than BDNF, could be an interesting biomarker candidate, and its dysregulation could be a general feature linked to neurodegeneration. Furthermore, it has been reported that, in contrast to BDNF, circulating Pro-BDNF is not released by platelet during activation, but seems to originate from other cell types [53]. Even though its origin remains an open question, it is possible that circulating levels are a more affordable mirror of neuronal health than those of BDNF.

To get a more complete picture, it would be important to measure the levels of Pro-BDNF in CSF. However, at the moment, this is not possible, due to the lack of a system of detection sensitive enough to measure this analyte at very low concentrations. Thus, we could not evaluate the Pro-BDNF role as a biomarker in CSF.

Due to the divergent behavior of circulating BDNF and Pro-BDNF in ALS compared to controls, their ratio (BDNF/Pro-BDNF) proved to have better diagnostic sensitivity and specificity than the individual markers. This is just a preliminary observation, which needs to be confirmed in a larger cohort of patients, and possibly in comparison with ALS-mimics conditions, before being considered for diagnostic purposes. It provides, though, interesting insights about the BDNF/Pro-BDNF balance in ALS. Of note, the same trend is also present in AD patients. This is not a big deal for a potential diagnostic use of these biomarkers, since AD is a condition usually not included in the differential diagnosis of ALS. Rather, it suggests that the pattern of BDNF/pro-BNDF, similar in ALS and AD patients, may be a typical feature of neurodegenerative diseases. However, while in all AD patients, serum levels of BDNF were increased and Pro-BDNF decreased compared to controls, in ALS patients, the trend showed some differences, with only approximately 63% of cases displaying the same trend as AD, suggesting a certain heterogeneity degree.

When BDNF and Pro-BDNF levels were evaluated in subgroups of ALS patients, stratified by clinical and genetic features, a significant difference between *C9orf72* expansion carriers and non-carriers emerged. BDNF levels in expansion-carrying patients were significantly lower than those of non-carrier patients and substantially comparable to those of the control subjects. Moreover, the proportion of subjects with a reduction in the levels of both BDNF and Pro-BDNF was four times higher in the *C9orf72* expansion carriers than in non-carriers (58.4% vs. 14.7%). This suggests that the presence of *C9orf72* expansion could be related to pathogenic mechanisms that have a peculiar impact on BDNF-mediated pathways. Interestingly, it has been recently reported that, in motor neurons derived from *C9orf72* patients, impaired endocytosis of the BDNF receptor TrkB negatively affects neuronal survival [54]. In neurons with decreased *C9orf72* expression, endocytosis of TrkB receptors is reduced, and this could affect signaling cascades regulating neuronal survival [55]. Thus, the alteration of TrkB receptors could modify the response to BDNF in *C9orf72* expansion carriers.

These observations should be considered when neurotrophins are evaluated as potential biomarkers of ALS. In patients carrying the *C9orf72* expansion, the quantification of serum BDNF levels is probably not appropriate and risks being a confounding factor for the evaluation and use of these biomarkers.

In our study, BDNF levels in CSF were significantly higher in the group of patients with fast progressive disease. Of note, all patients showing a CSF BDNF value greater than 0.7 pg/mL belonged to the fast progressive group when compared to patients with lower levels of BDNF. This observation may appear counterintuitive, since the BDNF capability to promote neuronal survival and resistance to toxic insults by its interaction with the TrkB receptor is well known [18]. The neuroprotective effects of BDNF on glutamate induced excitotoxicity have been demonstrated in vivo [56,57] and in vitro [58]. However, many studies have reported that BDNF/TrkB can exert negative effects on survival of motor neurons, making them more vulnerable to insults, mainly to the excitotoxic insult [59,60,61]. The activation of glutamate receptors, clearly observed in ALS patients, could enhance BDNF production, which in turn could trigger a further release of glutamate, perturbing neuron activity, increasing glutamate excitotoxicity and resulting in neuronal death [4]. Another possible explanation is related to BDNF receptors. Besides the full-length TrkB receptor, a truncated TrkB.T1 isoform with decreased tyrosine residue phosphorylation levels and dominant negative function has been described [62]. TrkB.T1 is expressed in all spinal cord cell populations, and its presence may limit BDNF effects in motor neurons, resulting in positive feedback that causes an increase in BDNF levels in CSF, while its cellular activity is decreased or impaired [62].

Furthermore, lower circulating Pro-BDNF levels were associated with a shorter survival in ALS patients. This finding may be quite surprising, since pro-BDNF has usually been considered as a proapoptotic mediator in neuronal cells [28] and a “punishment signal” during synaptic competition at the developing neuromuscular junctions [13]. However, while the pro-survival action of BDNF on central and peripheral neurons has been amply reported, the effect of Pro-BDNF on neuronal development and survival is less clear, and probably cell-type specific [63]. Pro-BDNF has been associated with neuronal apoptosis [24], but also with neuronal growth cone development, neuronal survival [23] and enhancement of the protective effect of BDNF [60]. Thus, the hypothesis of a protective role of Pro-BDNF cannot be ruled out. On the other hand, it should be recorded that levels revealed in blood do not necessarily reflect what happens in the central nervous system, and Pro-BDNF is not measurable in the CSF. In addition, serum Pro-BDNF levels could also differ from the real activity in the tissues, in particular in neuromuscular junctions. Thus, an increase in the Pro-BDNF (or rather a smaller decrease compared to the value of the controls) could reflect its lower use in tissues and neurons.

The present study has some limitations and strengths. Regarding the individuals enrolled in the study, a strength is that all ALS patients were clinically and genetically characterized and followed up. We compared the results with three control groups, consisting of a neurodegenerative disease group, an inflammatory disease group and a healthy subject group. The overall sample size gave a high statistical power; however, when ALS patients were stratified based on clinical variables, their number for each subgroup became smaller and the power decreased. Thus, correlations with clinical variables must be confirmed in a larger cohort of patients. Regarding the experimental approach, a strength of our study is certainly the dosage of both BDNF and Pro-BDNF. The opportunity to distinguish between the two molecules allows for obtaining more accurate results and better evaluating their changes. Moreover, a high sensitivity technology was used to measure BDNF levels in the CSF. Unfortunately, though, pro-BDNF is not quantifiable in the CSF yet, and this limits the overall understanding of BDNF/Pro-BDNF role in ALS. Further improvement of technology may allow more complete results to be obtained and may deepen our knowledge of the BDNF/Pro-BDNF balance in this disease.

## 5. Conclusions

We found that the serum levels of BDNF were increased in ALS patients compared to the control group, while serum Pro-BDNF concentrations were decreased. The use of the BDNF/Pro-BDNF ratio showed a high accuracy in distinguishing ALS patients from healthy controls, suggesting that it could represent a promising biomarker for ALS. Moreover, we provided evidence of interesting associations with ALS clinical variables. Patients carrying the *C9orf72* expansion significantly differed from non-carrier patients and showed circulating BDNF levels comparable to those of the control subjects. This finding may provide important insights about the pathogenesis of the disease and eventually contribute to better understanding the mechanisms mediated by BDNF in ALS. Furthermore, the BDNF levels in CSF were significantly higher in ALS patients with faster disease progression. Finally, lower serum levels of Pro-BDNF were associated with a shorter survival. All these data suggest that BDNF and Pro-BDNF, alone or in combination, may represent sensitive indicators of ALS progression and might be used as prognostic biomarkers. This could improve stratification of patients into subpopulations, prediction of prognosis, evaluation of treatment response and, eventually, might enhance clinical studies to develop personalized approaches to ALS.

## Figures and Tables

**Table 1:** Characterization of patients/controls participating in the study.

*SERUM*	ALS *n* = 75	AD *n* = 19	ID *n* = 20 (12 + 8) *	CTR *n* = 49
Mean age	64.1 ± 10.1	66.8 ± 7.4	44.7 ± 17.8 **	60.4 ± 10.8
(range)	37–87 y	52–77 y	19–85 y	42–89 y
Sex	M 36 (48.0%)	M 6 (31.6%)	M 11 (55.0%)	M 23 (47%)
(male/female)	F 39 (52.0%)	F 13 (68.4%)	F 9 (45.0%)	F 26 (53%)
***CSF***	**ALS** ***n* = 34**	**AD** ***n* = 10**	**ID** ***n* = 19**	**CTR** ***n* = 11**
Mean age	63.3 ± 8.8	66.9 ± 8.8	44.3 ± 21.5	60.3 ± 18.7
(range)	39–80 y	52–77 y	19–85 y	39–80 y
Sex	M 17 (50.0%)	M 3 (30.0%)	M 10 (52.6%)	M 4 (36.4%)
(male/female)	F 17 (50.0%)	F 7 (70.0%)	F 9 (47.4%)	F 7 (63.6%)

ALS: Amyotrophic Lateral Sclerosis; AD: Alzheimer Disease; ID: Inflammatory Diseases; CTR: controls; CSF: cerebral spinal fluid; M: male; F: female. * 12 patients with Multiple Sclerosis and 8 with Guillain-Barrè Syndrome. **: for the ID group, the mean age was significantly lower, mainly due to the MS group.

**Table 2:** Clinical variables of ALS patients.

ALS Patients	*n* = 75
Site of onset (spinal/bulbar)	S 60 (80%)/B 15 (20%)
Genetics (*C9orf72*+)	12 (16.0%)
Median BMI	24.82 ± 3.98
	(<25: 52.5%; >25: 47.5%)
Onset-diagnosis period	13.1 ± 8.9 months
Disease duration	37.2 ± 21.9 months

S: spinal; B: bulbar; BMI: Body Mass Index.

**Table 3:** BDNF/Pro-BDNF ratio in ALS, AD, ID patients and control subjects.

	BDNF/Pro-BDNF	*p* Value (Compared to CTR)
CTR	0.88 ± 0.134	
ALS	2.53 ± 0.384	<0.00001
AD	4.93 ± 0.479	<0.00001
ID	1.18 ± 0.146	0.02729

**Table 4:** Associations of serum BDNF and Pro-BDNF levels with clinical variables of ALS patients.

	Sex	Site of Onset	Age of Onset *	*C9orf72*	BMI	Progression Rate
BDNF *p* value	0.889	0.373	0.306	**0.026**	0.976	0.354
Pro-BDNF *p* value	0.352	0.276	0.593	0.749	**0.035**	0.534

* Spearman’s rank. *p* values statistically significant are shown in bold.

**Table 5:** Associations with ALS clinical variables stratifying ALS patients based on BDNF and Pro-BDNF trends versus controls.

	Sex		Site of Onset		Age of Onset		*C9orf72* Expansion		BMI		Progression Rate	
	M	F	S	B	<45 y	>45 y	*C9orf72*+	*C9orf72*−	<25	>25	Fast	S + I
**↑B ↑P**	13.9	15.4	15.0	13.3	40.0	12.9	8.3	14.8	18.2	5.3	14.3	16.0
**↑B ↓P**	72.2	56.4	66.7	53.4	40.0	65.7	33.3	70.5	45.4	63.2	57.1	68.0
**↓B ↓P**	13.9	28.2	18.3	33.3	20.0	21.4	58.4	14.7	36.4	31.5	28.6	16.0
***p* value**	0.275		0.669		0.462		**0.007**		0.452		0.639	

All data in the table are reported as percentages. B: BDNF; P: Pro-BDNF; ↑: increase; ↓: decrease (defined comparing BDNF and Pro-BDNF levels to the median values of the control group). *p* values statistically significant are shown in bold.

**Table 6:** Associations of CSF BDNF levels with ALS clinical variables.

	Sex	Site of Onset	Age of Onset ¹	*C9orf72*	BMI	Progression Rate	Survival ^2^
*p* value	0.522	0.104	0.445	0.813	0.984	**0.026**	0.758

¹ Spearman’s rank. ^2^ Kaplan-Meier log-rank. *p* values statistically significant are shown in bold.

## Data Availability

The data presented in this study are available on request from the corresponding author. The data are not publicly available due to privacy restrictions.

## Supplementary Materials

The following supporting information can be downloaded at: https://www.mdpi.com/article/10.3390/brainsci12050617/s1, Table S1: BDNF and Pro-BDNF levels in the two different subgroups of Inflammatory Diseases; Table S2: BDNF/Pro-BDNF ratio in subgroups of Inflammatory Diseases; Figure S1: Correlation between CSF BDNF and serum BDNF levels in GBS patients; Figure S2: Correlation between CSF BDNF and serum Pro-BDNF levels in GBS patients; Figure S3: Receiver operating characteristic (ROC) curve calculated for BDNF, 1-Pro-BDNF and BDNF/Pro-BDNF; Table S3: Associations of serum/CSF BDNF and serum Pro-BDNF levels with ALSFRS-R and FVC of ALS patients; Table S4: Associations with ALSFRS-R and FVC stratifying ALS patients based on BDNF and Pro-BDNF trends versus controls; Figure S4: Kaplan-Meier survival curves in relation to BDNF and Pro-BDNF levels considering all ALS patients.
