# Supplementary material for: BDNF and Pro-BDNF in Amyotrophic Lateral Sclerosis: A New Perspective for Biomarkers of Neurodegeneration
Source: Brain Sci. 2022 May 9;12(5):617. doi: 10.3390/brainsci12050617 (PMC9139087; doi:10.3390/brainsci12050617)
Supplement: Supplementary file 1 [file brainsci-12-00617-s001.zip › toxins-1659474-supplementary.pdf]

Supplementary file

BDNF and Pro-BDNF in Amyotrophic Lateral Sclerosis: new perspective for biomarkers of neurodegeneration

Giulia Riolo, Claudia Ricci, Nicoletta De Angelis, Carlotta Marzocchi, Gisella Guerrera, Giovanna Borsellino, Fabio Giannini and Stefania Battistini

Table S1. BDNF and Pro-BDNF levels in the two different subgroups of Inflammatory Diseases.

	BDNF (pg/ml)	MS vs. CTR	GBS vs. CTR	MS vs. GBS	Pro-BDNF (pg/ml)	MS vs. CTR	GBS vs. CTR	MS vs. GBS
**CTR**	9098.68±1210.76				12522.61±885.46			
**MS**	15971.33±1296.11				12063.58±1307.60			
**GBS**	16493.87±2811.74				25266.75±5948.06			
**p value**		**0.026**	**0.033**	0.960		0.922	**<0.0001**	**0.006**

CTR: controls; MS: Multiple Sclerosis; GBS: Guillan-Barré Syndrome.

Due to the Pro-BDNF differences between MS and GBS patients, BDFN/Pro-BDNF ratio showed a different trend in the two subgroups of Inflammatory Diseases (ID), as summarized in **Supplementary Table 2**.

Table S2. BDNF/Pro-BDNF ratio in soubgroups od Inflammatory Diseases

	p value
ID vs. CTR	**0.027**
MS vs. CTR	**0.016**
GBS vs. CTR	0.453
MS vs. GBS	**0.008**

Of note, in GBS patients there was a positive correlation between CSF BDNF levels and both serum BDNF and Pro-BDNF levels (p=0.028 and p=0.037, respectively), which was not present in MS patients (p=0.351 and p=0.555, respectively).

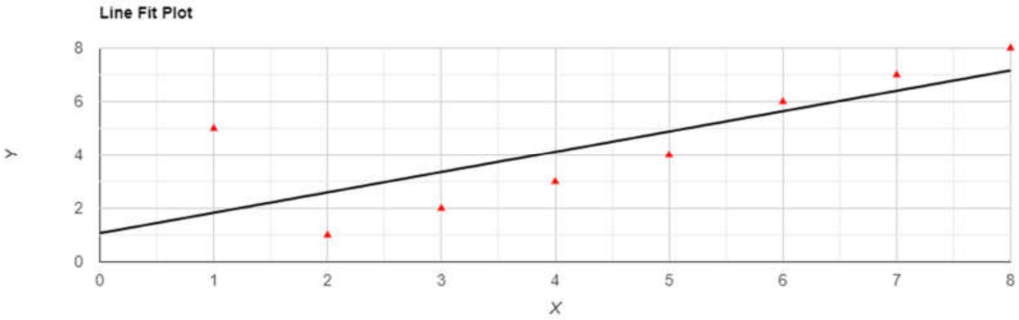

Figure S1. Correlation between CSF BDNF and serum BDNF levels in GBS patients. $r_s = 0.7619$, p=0.028

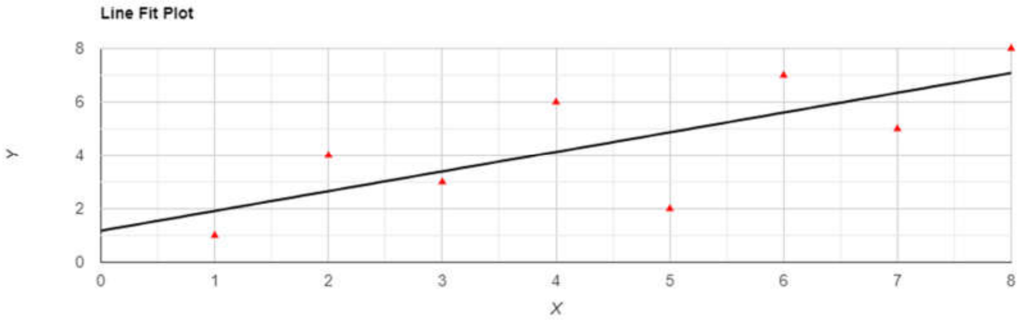

Figure S2. Correlation between CSF BDNF and serum Pro-BDNF levels in GBS patients. $r_s = 0.7381$, p= 0.037

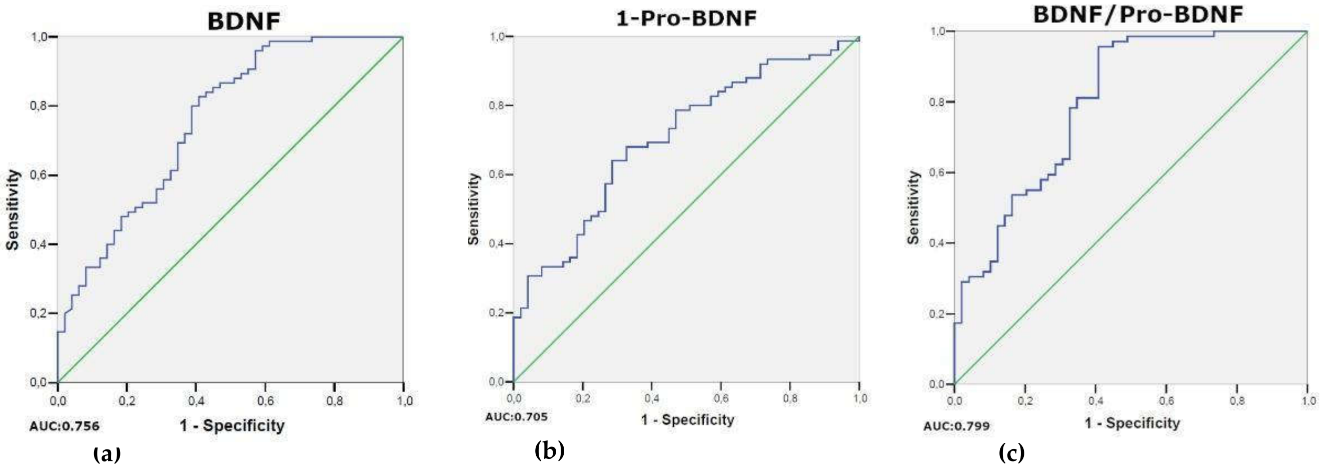

Figure S3. Receiver operating characteristic (ROC) curve calculated for (a) BDNF, (b) 1-Pro-BDNF, and (c) BDNF/Pro-BDNF. BDNF/Pro-BDNF ratio showed the highest accuracy in discriminating ALS patients from controls. AUC: area under curve.

Table S3. Associations of serum/CSF BDNF and serum Pro-BDNF levels with ALSFRS-R and FVC of ALS patients.

	ALSFRS-R	FVC
Serum BDNF *p value*	0.152	0.636
Serum Pro-BDNF *p value*	0.151	0.864
CSF BDNF *p value*	0.357	0.733

ALSFRS-R: Revised Amyotrophic Lateral Sclerosis Functional Rating Scale; FVC: Forced Vital Capacity

Table S4. Associations with ALSFRS-R and FVC stratifying ALS patients based on BDNF and Pro-BDNF trends versus controls.

	ALSFRS-R	FVC
**↑B ↑P**	12.5	11.1
**↑B ↓P**	56.3	61.1
**↓B ↓P**	31.2	27.8
p value	0.851	0.592

All data in the Table are reported as percentages. ALSFRS-R: Revised Amyotrophic Lateral Sclerosis Functional Rating Scale; FVC: Forced Vital Capacity. ↑: increase; ↓: decrease (defined comparing BDNF and Pro-BDNF levels to the median values of the control group).

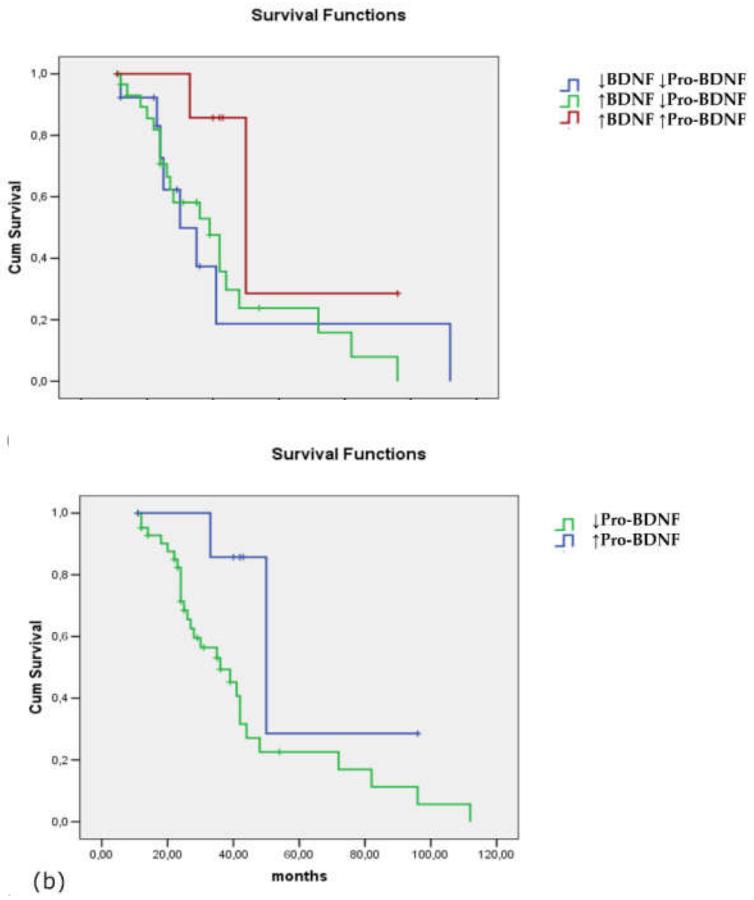

Figure S4. Kaplan-Meier survival curves in relation to BDNF and Pro-BDNF levels considering all ALS patients. (a) Blue line: decrease of both BDNF and Pro-BDNF levels; green line: increased BDNF and decreased Pro-BDNF; burgundy line: increase of both BDNF and Pro-BDNF levels; ($p=0.149$). **(b)** Green line: decreased Pro-BDNF; blue line: increased pro-BDNF levels (regardless BDNF levels); ($p=0.051$). **(c)** Censored values (+) indicate the last known follow-up time for those subjects still alive at the time of analysis.
